# Crystal structure of 5-chloro­methyl-*N*-methyl-4-[(4-phenyl-1,2,3-triazol-1-yl)meth­yl]isoxazolidine-3-carboxamide

**DOI:** 10.1107/S2056989016002784

**Published:** 2016-02-20

**Authors:** Jihed Brahmi, Soumaya Nasri, Kaïss Aouadi, Erwann Jeanneau, Sébastien Vidal, Moncef Msaddek

**Affiliations:** a Université de Monastir, Laboratoire de Synthèse Hétérocyclique, Produits Naturels et Réactivités, Faculté des Sciences de Monastir, Avenue de l’Environnement, 5000 Monastir, Tunisia; b Laboratoire de Physico-chimie des Matériaux, Faculté des Sciences de Monastir, Avenue de l’Environnement, 5019 Monastir, University of Monastir, Tunisia; c Université Lyon 1, Centre de Diffractométrie Henri Longchambon Bâtiment 305, 43 Boulevard du 11 Novembre 1918, F-69622 Villeurbanne Cedex, France; d Université de Lyon, Institut de Chimie et Biochimie Moléculaires et Supramoléculaires, UMR CNRS 5246, Laboratoire de Chimie Organique 2-Glycochimie, Bâtiment Curien, 43 boulevard du 11 Novembre 1918, F-69622 Villeurbanne, France

**Keywords:** crystal structure, 1,3-dipolar cyclo­addition, isoxazolidine, hydrogen bonding

## Abstract

The title compound, C_15_H_18_ClN_5_O_2_, crystallizes with two independent mol­ecules (*A* and *B*) in the asymmetric unit. In both mol­ecules, the isoxazolidine rings have an envelope conformation with the O atoms at the flap positions. Each mol­ecule has three stereogenic centres with configurations 2(*S*), 3(*S*) and 4(*R*), confirmed by resonant scattering. Their conformations are significantly different, for example in mol­ecule *A* the phenyl ring is inclined to the triazole ring by 32.5 (2)°, while in mol­ecule *B* the corresponding dihedral angle is 10.7 (2)°. In the crystal, the *A* and *B* mol­ecules are linked *via* an N—H⋯O and a C—H⋯O hydrogen bond. These units are linked by C—H⋯O and C—H⋯N hydrogen bonds, forming slabs parallel to the *ab* plane. There are C—H⋯π inter­actions present within the slabs.

## Abstract 2

The title compound crystallized with two independent mol­ecules in the asymmetric unit. Each mol­ecule has three stereogenic centres with configurations 2(*S*), 3(*S*) and 4(*R*), confirmed by resonant scattering.

## Chemical context   

The 1,3-dipolar cyclo­addition of nitro­nes to alkenes provides a straightforward route to isoxazolidines (Frederickson, 1997 ▸; Gothelf *et al.*, 2002 ▸). Nitrone cyclo­adducts are attractive inter­mediates for the synthesis of several classes of natural products and biologically active compounds, such as unnatural amino­acids (Aouadi, *et al.*, 2006 ▸) and alkaloids; for example (+)-febrifugine, (−)-indolizidine 209B (Smith *et al.*, 1988 ▸), (+)-sedridine (Louis & Hootelé, 1995 ▸, 1997 ▸; Huisgen, 1984 ▸). We report herein on the synthesis, the mol­ecular structure and the spectroscopic data of the title compound, (**2**).
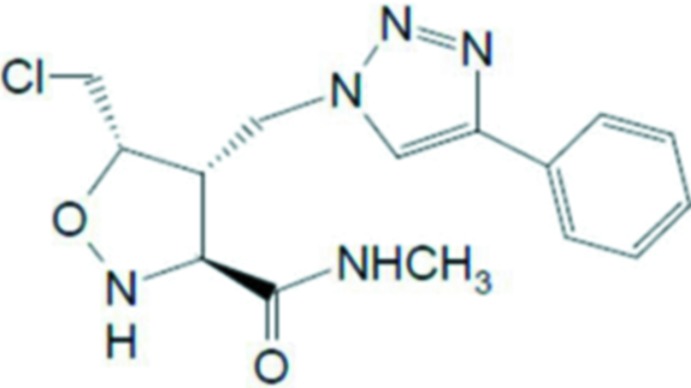


## Structural commentary   

**Figure 1:**
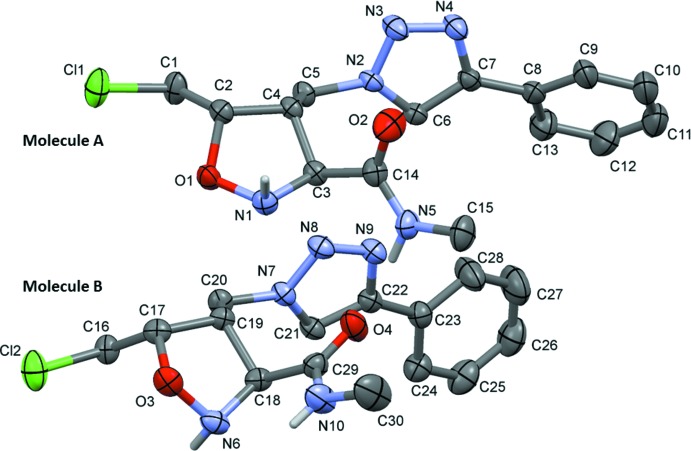
The mol­ecular structure of the two independent mol­ecules of compound (**2**), showing the atom labelling. Displacement ellipsoids are drawn at the 30% probability level. C-bound H atoms have been omitted for clarity.

The title compound (**2**), Fig. 1 ▸, crystallized in the non-centrosymmetric space group *P2_1_*, with two independent mol­ecules (*A* and *B*) in the asymmetric unit. Each mol­ecule has three stereogenic centres with configurations 2(*S*), 3(*S*) and 4(*R*), confirmed by resonant scattering [Flack parameter = −0.012 (6)]. In mol­ecule *B* there is an intra­molecular N—H⋯N contact present (Table 1 ▸).

**Figure 2:**
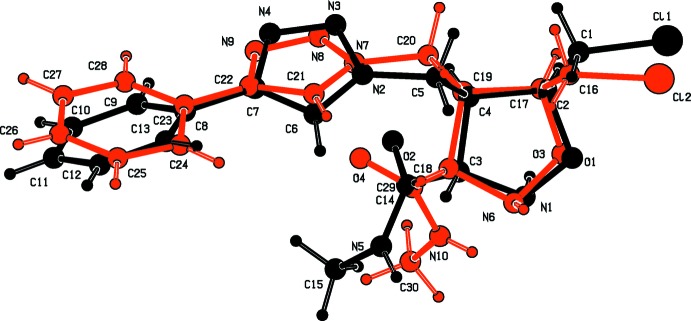
*AutoMolFit* (Spek, 2009 ▸) of the two independent mol­ecules (*A* black, *B* red) of compound (**2**).

The conformations of the two mol­ecules differ significantly, as seen in the overlay fit of the two mol­ecules (Fig. 2 ▸). In mol­ecule *A* the phenyl ring is inclined to the triazole ring by 32.5 (2)°, while in mol­ecule *B* the corresponding dihedral angle is 10.7 (2)°. The torsion angle C6—C7—C8—C13 is 31.5 (5)° in mol­ecule *A*, while torsion angle C21—C22—C23—C24, is −9.0 (5)° in mol­ecule *B*. The isoxazolidine rings (O1/N1/C2–C4 in mol­ecule *A* and O3/N6/C17–C19 in mol­ecule *B*) adopt envelope conformations. In mol­ecule *A* atom O1 is displaced by 0.566 (2) Å from the mean plane through atoms N1/C2–C4, while in mol­ecule *B* atom O3 is displaced by 0.528 (2) Å from the mean plane through atoms N6/C17–C19. Their mean planes are inclined to the relevant triazole ring by 53.95 (19)° in mol­ecule *A* and by 62.32 (18)° in mol­ecule B.

The triazole N—N distances N2—N3 and N3—N4 in mol­ecule *A* are 1.340 (4) and 1.307 (4) Å, respectively, and in mol­ecule *B* distances N7—N8 and N8—N9 are 1.346 (3) and 1.305 (4) Å, respectively. They are close to the values reported for related triazole compounds, for example 2-allyl-3-[(1-benzyl-1*H*-1,2,3-triazol-4-yl)meth­oxy]-4-meth­oxy­phenol (Chang *et al.*, 2014 ▸), with distances 1.357 (9) and 1.336 (7) Å. The N—O bond lengths of the isoxazolidine rings are O1—N1 = 1.442 (3) Å in *A* and O3—N6 = 1.445 (4) Å in *B*, also close to values reported for related compounds (Lee *et al.*, 2010 ▸; Molander & Cavalcanti, 2013 ▸).

## Supra­molecular features   

**Figure 3:**
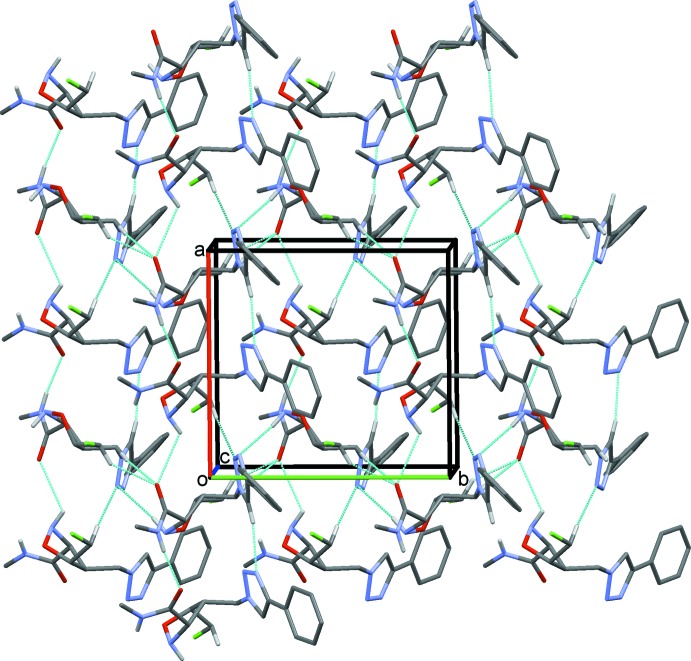
A view along the *c* axis of the crystal packing of compound (**2**). Hydrogen bonds are shown as dashed lines (see Table 1 ▸) and H atoms not involved in these inter­actions have been omitted for clarity.

In the crystal of (**2**), the two independent mol­ecules are linked *via* an N—H⋯O and a C—H⋯O hydrogen bond (Table 1 ▸ and Fig. 3 ▸). These units are then linked *via* C—H⋯O and C—H⋯N hydrogen bonds, forming slabs lying parallel to the *ab* plane (Table 1 ▸ and Fig. 3 ▸). Within the slabs there are C—H⋯π inter­actions present involving symmetry-related *A* mol­ecules (Table 1 ▸).

## Synthesis and crystallization   

**Figure 4:**
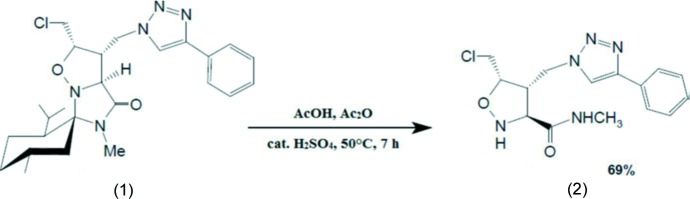
Reaction scheme.

The title compound, (**2**), was synthesized in two steps. Starting with a 1,3-dipolar cyclo­addition between (1*S*,2*S*,5*S*)-3′-(azido­meth­yl)-2′-(cholormeth­yl)-2-isopropyl-5,5′-di­methyl­dihydro-5′*H*-spiro­[cyclo­hexane-1,6′-imidazo[1,5-*b*]isoxasol]-4′(5′*H*)-one and phenyl­acetyl­ene lead to the formation of 1,2,3-triazolyl-functionalized isoxazolidine, compound (**1**) [yield 88%]. The cyclo­adduct (**1**) (200 mg, 0.42 mmol) was then dissolved in Ac_2_O (2 ml), AcOH (3 ml), concentrated H_2_SO_4_ (0.8 ml) and the reaction was stirred at 323 K for 7 h. After cooling to 273 K, an aqueous solution of 5% NaOH was added drop wise over a period of 2 h until pH 8. The mixture was then poured slowly into a saturated aqueous NaHCO_3_ solution (280 ml). The resulting mixture was extracted with CH_2_Cl_2_ (3 × 100 ml) and the combined organic phases were dried with Na_2_SO_4_. After filtration and evaporation of the solvents under reduced pressure, the residue was purified by flash chromatography (silica gel: EtOAc/PE, 8:2) to afford the desired title compound (**2**) as a white solid (97 mg, yield 69%); see Fig. 4 ▸. Colourless block-like crystals of (**2**) were obtained by slow evaporation of a solution in di­chloro­methane.

## Spectroscopic investigations   

The spectroscopic measurements are consistent with the crystal structure of (**2**). High-resolution mass spectrometry in positive-ion mode gave an [*M* + H]^+^ ion of 336.1221 *m*/*z*, close to the calculated mass of 336.1222 *m*/*z*. The ^1^H NMR spectrum of (**2**) shows the presence the triazole ring proton at 7.96 p.p.m. The ^13^C NMR spectrum confirms the existence of the three, C2, C3 and C4, stereogenic centres (80.3 p.p.m., 64.2 p.p.m. and 48.4 p.p.m., respectively).

*R*_f_ = 0.58 [EtOAc/PE 9/1]. NMR ^1^H (400 MHz, CDCl_3_): δ(p.p.m.): 2.81 (*d*, 3H, CH_3_, *J* 4.0 Hz), 3.49 (*quin*, 1H, *J* 5.6 Hz), 3.84 (*dd*, 1H, *J* 4.0, 9.6 Hz), 3.92 (d, 1H, *J* 4.4 Hz), 4.04 (*dd*, 1H, *J* 3.6, 9.6 Hz), 4.46 (*dd*, 1H, *J* 4.4, 9.6 Hz), 4.89 (*m*, 2H), 7.35 (*t*, 1H, *J* 6.0 Hz), 7.43 (*t*, 2H, *J* 6.0 Hz), 7.81 (*d*, 2H, *J* 5.6 Hz), 7.96 (*s*, 1H triazole). NMR ^13^C (100 MHz, CDCl_3_): δ(p.p.m.): 30.9, 42.2, 48.4, 50.9, 64.2, 80.3, 120.4, 125.7, 128.5, 128.9, 130.0, 148.4, 170.6 (C=O). HRMS, (ESI) calculated C_15_H_19_ClN_5_O_2_ [*M* + H^+^] = 336.1222, found: 336.1221. [α]^22^ = + 32.6 (*c* = 1; CH_2_Cl_2_).

## Refinement   

Crystal data, data collection and structure refinement details are summarized in Table 2 ▸. The NH H atoms were located in a difference Fourier map and freely refined. The C-bound H atoms were fixed geometrically and treated as riding: C—H = 0.93–0.98 Å with *U*_iso_(H) = 1.2*U*_eq_(C).

## Supplementary Material

Crystal structure: contains datablock(s) I, global. DOI: 10.1107/S2056989016002784/su5281sup1.cif

Structure factors: contains datablock(s) I. DOI: 10.1107/S2056989016002784/su5281Isup2.hkl

Supporting information file. DOI: 10.1107/S2056989016002784/su5281Isup3.cml

CCDC reference: 1046833

Additional supporting information:  crystallographic information; 3D view; checkCIF report

## Figures and Tables

**Table 1:** Hydrogen-bond geometry (Å, °) *Cg*2 is the centroid of the triazole ring N2–N4/C6/C7 in mol­ecule *A*.

*D*—H⋯*A*	*D*—H	H⋯*A*	*D*⋯*A*	*D*—H⋯*A*
N10—H10*N*⋯N6	0.78 (4)	2.21 (4)	2.668 (4)	118 (4)
N5—H5*N*⋯O4	0.91 (4)	2.02 (4)	2.925 (4)	173 (4)
C6—H6⋯N9	0.93	2.37	3.275 (4)	164
C1—H1*B*⋯O2^i^	0.97	2.36	3.208 (4)	146
C5—H5*B*⋯O2^i^	0.97	2.47	3.432 (4)	171
C16—H16*A*⋯N3^ii^	0.97	2.37	3.335 (4)	176
C2—H2⋯*Cg*2^iii^	0.95	2.90	3.806 (3)	154

Symmetry codes: (i) 
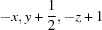
; (ii) 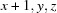; (iii) 
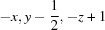
.

**Table 2:** Experimental details

Crystal data	
Chemical formula	C_15_H_18_ClN_5_O_2_
*M*_r_	335.79
Crystal system, space group	Monoclinic, *P*2_1_
Temperature (K)	293
*a*, *b*, *c* (Å)	10.8355 (2), 10.8865 (2), 14.5653 (2)
β (°)	106.481 (2)
*V* (Å^3^)	1647.54 (5)
*Z*	4
Radiation type	Cu *K*α
μ (mm^−1^)	2.20
Crystal size (mm)	0.36 × 0.34 × 0.17

Data collection	
Diffractometer	Agilent Xcalibur (Atlas, Gemini ultra)
Absorption correction	Analytical (*CrysAlis PRO*; Agilent, 2013 ▸)
*T*_min_, *T*_max_	0.518, 0.721
No. of measured, independent and observed [*I* > 2σ(*I*)] reflections	33842, 5824, 5486
*R*_int_	0.050
(sin θ/λ)_max_ (Å^−1^)	0.596

Refinement	
*R*[*F* ^2^ > 2σ(*F* ^2^)], *wR*(*F* ^2^), *S*	0.036, 0.098, 1.03
No. of reflections	5824
No. of parameters	431
No. of restraints	1
H-atom treatment	H atoms treated by a mixture of independent and constrained refinement
Δρ_max_, Δρ_min_ (e Å^−3^)	0.17, −0.25
Absolute structure	Flack *x* determined using 2460 quotients [(*I* ^+^)−(*I* ^−^)]/[(*I* ^+^)+(*I* ^−^)] (Parsons *et al.*, 2013 ▸)
Absolute structure parameter	−0.012 (6)

Computer programs: *CrysAlis PRO* (Agilent, 2013 ▸), *SIR2004* (Burla *et al.*, 2005 ▸), *SHELXL2014* (Sheldrick, 2015 ▸), *Mercury* (Macrae *et al.*, 2008 ▸), *WinGX* (Farrugia, 2012 ▸) and *PLATON* (Spek, 2009 ▸).

## supplementary crystallographic information

### Crystal data

**Table:**

C_15_H_18_ClN_5_O_2_	*F*(000) = 704
*M**_r_* = 335.79	*D*_x_ = 1.354 Mg m^−^^3^
Monoclinic, *P*2_1_	Cu *K*α radiation, λ = 1.54178 Å
*a* = 10.8355 (2) Å	Cell parameters from 16111 reflections
*b* = 10.8865 (2) Å	θ = 4.1–66.7°
*c* = 14.5653 (2) Å	µ = 2.20 mm^−^^1^
β = 106.481 (2)°	*T* = 293 K
*V* = 1647.54 (5) Å^3^	Block, colourless
*Z* = 4	0.36 × 0.34 × 0.17 mm

### Data collection

**Table:**

Agilent Xcalibur (Atlas, Gemini ultra) diffractometer	5824 independent reflections
Radiation source: Enhance Ultra (Cu) X-ray Source	5486 reflections with *I* > 2σ(*I*)
Detector resolution: 10.4678 pixels mm^-1^	*R*_int_ = 0.050
ω scans	θ_max_ = 66.7°, θ_min_ = 3.2°
Absorption correction: analytical (*CrysAlis PRO*; Agilent, 2013)	*h* = −12→12
*T*_min_ = 0.518, *T*_max_ = 0.721	*k* = −12→12
33842 measured reflections	*l* = −17→17

### Refinement

**Table:**

Refinement on *F*^2^	Secondary atom site location: difference Fourier map
Least-squares matrix: full	Hydrogen site location: mixed
*R*[*F*^2^ > 2σ(*F*^2^)] = 0.036	H atoms treated by a mixture of independent and constrained refinement
*wR*(*F*^2^) = 0.098	*w* = 1/[σ^2^(*F*_o_^2^) + (0.0615*P*)^2^ + 0.2009*P*] where *P* = (*F*_o_^2^ + 2*F*_c_^2^)/3
*S* = 1.02	(Δ/σ)_max_ < 0.001
5824 reflections	Δρ_max_ = 0.17 e Å^−^^3^
431 parameters	Δρ_min_ = −0.25 e Å^−^^3^
1 restraint	Absolute structure: Flack *x* determined using 2460 quotients [(*I*^+^)-(*I*^-^)]/[(*I*^+^)+(*I*^-^)] (Parsons *et al.*, 2013)
Primary atom site location: structure-invariant direct methods	Absolute structure parameter: −0.012 (6)

### Special details

**Table:**

Geometry. All e.s.d.'s (except the e.s.d. in the dihedral angle between two l.s. planes) are estimated using the full covariance matrix. The cell e.s.d.'s are taken into account individually in the estimation of e.s.d.'s in distances, angles and torsion angles; correlations between e.s.d.'s in cell parameters are only used when they are defined by crystal symmetry. An approximate (isotropic) treatment of cell e.s.d.'s is used for estimating e.s.d.'s involving l.s. planes.

### Fractional atomic coordinates and isotropic or equivalent isotropic displacement parameters (Å^2^)

**Table:**

	*x*	*y*	*z*	*U*_iso_*/*U*_eq_	
Cl1	0.13936 (13)	0.47609 (9)	0.24767 (6)	0.0825 (3)	
Cl2	0.73439 (12)	0.41058 (9)	0.36164 (7)	0.0820 (3)	
O1	0.2546 (2)	0.3689 (2)	0.45141 (15)	0.0517 (5)	
O2	0.0503 (3)	0.2666 (3)	0.6455 (2)	0.0865 (9)	
O3	0.6733 (2)	0.28927 (18)	0.53585 (18)	0.0581 (5)	
O4	0.5087 (3)	0.3654 (3)	0.7570 (2)	0.0739 (7)	
N1	0.2650 (3)	0.2979 (3)	0.53684 (19)	0.0517 (6)	
H1N	0.205 (4)	0.247 (4)	0.514 (3)	0.055 (11)*	
N2	0.0602 (2)	0.6113 (2)	0.63212 (17)	0.0419 (5)	
N3	−0.0647 (3)	0.5920 (3)	0.6239 (2)	0.0570 (7)	
N4	−0.0824 (2)	0.6133 (3)	0.7076 (2)	0.0562 (7)	
N5	0.2516 (3)	0.2708 (3)	0.7437 (2)	0.0620 (7)	
H5N	0.334 (4)	0.295 (4)	0.751 (3)	0.064 (11)*	
N6	0.7486 (3)	0.3170 (3)	0.6325 (2)	0.0555 (6)	
H6N	0.818 (4)	0.364 (4)	0.628 (3)	0.068 (11)*	
N7	0.5466 (2)	0.6450 (2)	0.67354 (17)	0.0437 (5)	
N8	0.4305 (2)	0.6292 (2)	0.68824 (19)	0.0511 (6)	
N9	0.4348 (2)	0.6829 (3)	0.76918 (19)	0.0517 (6)	
N10	0.6278 (4)	0.2017 (3)	0.7438 (3)	0.0762 (10)	
H10N	0.685 (4)	0.183 (4)	0.724 (3)	0.060 (12)*	
C1	0.1254 (4)	0.5279 (3)	0.3610 (2)	0.0621 (9)	
H1A	0.1957	0.5835	0.3897	0.075*	
H1B	0.0453	0.5727	0.3514	0.075*	
C2	0.1281 (3)	0.4226 (3)	0.4275 (2)	0.0432 (6)	
H2	0.0650	0.3611	0.3947	0.052*	
C3	0.2096 (3)	0.3809 (2)	0.5948 (2)	0.0431 (6)	
H3	0.2765	0.4371	0.6309	0.052*	
C4	0.1038 (3)	0.4550 (2)	0.52417 (19)	0.0406 (6)	
H4	0.0199	0.4219	0.5246	0.049*	
C5	0.1076 (3)	0.5915 (3)	0.5481 (2)	0.0428 (6)	
H5A	0.1952	0.6217	0.5614	0.051*	
H5B	0.0545	0.6367	0.4938	0.051*	
C6	0.1227 (3)	0.6479 (3)	0.7208 (2)	0.0423 (6)	
H6	0.2093	0.6685	0.7443	0.051*	
C7	0.0312 (3)	0.6486 (3)	0.7694 (2)	0.0452 (6)	
C8	0.0431 (3)	0.6797 (3)	0.8698 (2)	0.0544 (8)	
C9	−0.0314 (4)	0.6204 (5)	0.9194 (3)	0.0740 (11)	
H9	−0.0916	0.5621	0.8883	0.089*	
C10	−0.0172 (5)	0.6469 (6)	1.0144 (3)	0.0953 (16)	
H10	−0.0682	0.6065	1.0465	0.114*	
C11	0.0695 (6)	0.7304 (6)	1.0614 (3)	0.0981 (17)	
H11	0.0791	0.7470	1.1257	0.118*	
C12	0.1425 (5)	0.7898 (6)	1.0141 (3)	0.1006 (16)	
H12	0.2021	0.8478	1.0464	0.121*	
C13	0.1303 (4)	0.7658 (5)	0.9179 (3)	0.0772 (11)	
H13	0.1809	0.8079	0.8865	0.093*	
C14	0.1629 (3)	0.3015 (3)	0.6648 (2)	0.0510 (7)	
C15	0.2257 (5)	0.1867 (4)	0.8142 (3)	0.0798 (12)	
H15A	0.3024	0.1763	0.8663	0.120*	
H15B	0.1586	0.2200	0.8380	0.120*	
H15C	0.1990	0.1086	0.7847	0.120*	
C16	0.6835 (3)	0.4838 (3)	0.4541 (2)	0.0504 (7)	
H16A	0.7586	0.5112	0.5038	0.060*	
H16B	0.6328	0.5557	0.4280	0.060*	
C17	0.6038 (3)	0.3994 (3)	0.4983 (2)	0.0475 (6)	
H17	0.5246	0.3770	0.4492	0.057*	
C18	0.6645 (3)	0.3930 (3)	0.6704 (2)	0.0460 (6)	
H18	0.7162	0.4560	0.7121	0.055*	
C19	0.5684 (3)	0.4565 (2)	0.5844 (2)	0.0412 (6)	
H19	0.4811	0.4305	0.5823	0.049*	
C20	0.5741 (3)	0.5967 (3)	0.5880 (2)	0.0465 (6)	
H20A	0.6591	0.6235	0.5869	0.056*	
H20B	0.5121	0.6297	0.5316	0.056*	
C21	0.6248 (3)	0.7090 (3)	0.7458 (2)	0.0465 (6)	
H21	0.7097	0.7316	0.7526	0.056*	
C22	0.5530 (3)	0.7341 (3)	0.8071 (2)	0.0460 (6)	
C23	0.5844 (3)	0.8079 (3)	0.8951 (2)	0.0516 (7)	
C24	0.7070 (4)	0.8516 (3)	0.9358 (2)	0.0605 (8)	
H24	0.7733	0.8293	0.9104	0.073*	
C25	0.7319 (5)	0.9290 (4)	1.0149 (3)	0.0801 (12)	
H25	0.8147	0.9595	1.0407	0.096*	
C26	0.6387 (6)	0.9607 (5)	1.0550 (3)	0.0882 (13)	
H26	0.6572	1.0113	1.1086	0.106*	
C27	0.5183 (6)	0.9182 (7)	1.0163 (4)	0.1109 (19)	
H27	0.4532	0.9412	1.0429	0.133*	
C28	0.4899 (5)	0.8403 (6)	0.9372 (3)	0.0947 (16)	
H28	0.4069	0.8100	0.9126	0.114*	
C29	0.5929 (3)	0.3181 (3)	0.7277 (2)	0.0535 (7)	
C30	0.5727 (6)	0.1189 (5)	0.7996 (5)	0.112 (2)	
H30A	0.6119	0.0394	0.8020	0.168*	
H30B	0.5879	0.1505	0.8633	0.168*	
H30C	0.4817	0.1119	0.7701	0.168*	

### Atomic displacement parameters (Å^2^)

**Table:**

	*U*^11^	*U*^22^	*U*^33^	*U*^12^	*U*^13^	*U*^23^
Cl1	0.1368 (9)	0.0649 (5)	0.0552 (4)	0.0136 (6)	0.0425 (5)	−0.0007 (4)
Cl2	0.1252 (9)	0.0624 (5)	0.0743 (5)	−0.0052 (5)	0.0539 (6)	−0.0140 (4)
O1	0.0530 (12)	0.0532 (12)	0.0547 (11)	0.0096 (9)	0.0248 (9)	0.0016 (9)
O2	0.0750 (18)	0.092 (2)	0.0879 (19)	−0.0397 (16)	0.0165 (14)	0.0207 (16)
O3	0.0695 (14)	0.0334 (10)	0.0765 (14)	0.0012 (10)	0.0289 (11)	−0.0027 (10)
O4	0.0809 (17)	0.0718 (16)	0.0832 (16)	0.0124 (13)	0.0463 (14)	0.0203 (13)
N1	0.0594 (16)	0.0423 (13)	0.0569 (14)	0.0089 (13)	0.0222 (12)	0.0025 (11)
N2	0.0374 (12)	0.0398 (12)	0.0513 (12)	0.0022 (9)	0.0169 (10)	−0.0050 (10)
N3	0.0412 (14)	0.0680 (17)	0.0646 (15)	−0.0060 (12)	0.0199 (12)	−0.0174 (13)
N4	0.0444 (14)	0.0663 (17)	0.0634 (15)	−0.0047 (12)	0.0244 (12)	−0.0107 (13)
N5	0.0652 (19)	0.0701 (19)	0.0548 (15)	−0.0044 (15)	0.0235 (13)	0.0141 (13)
N6	0.0457 (14)	0.0476 (14)	0.0754 (17)	0.0045 (12)	0.0207 (13)	0.0107 (13)
N7	0.0459 (13)	0.0361 (11)	0.0530 (12)	0.0002 (10)	0.0202 (10)	−0.0014 (10)
N8	0.0416 (13)	0.0498 (14)	0.0630 (15)	−0.0039 (11)	0.0165 (11)	−0.0046 (12)
N9	0.0444 (14)	0.0527 (14)	0.0618 (15)	−0.0016 (11)	0.0211 (11)	−0.0035 (12)
N10	0.076 (2)	0.0606 (19)	0.103 (3)	0.0159 (16)	0.043 (2)	0.0395 (18)
C1	0.095 (3)	0.0444 (16)	0.0530 (16)	0.0131 (17)	0.0312 (17)	0.0003 (14)
C2	0.0448 (15)	0.0368 (13)	0.0492 (14)	−0.0011 (11)	0.0154 (11)	−0.0054 (11)
C3	0.0460 (15)	0.0358 (13)	0.0503 (14)	−0.0021 (11)	0.0180 (12)	−0.0012 (11)
C4	0.0397 (13)	0.0361 (14)	0.0488 (13)	−0.0022 (10)	0.0171 (11)	−0.0062 (11)
C5	0.0507 (16)	0.0337 (13)	0.0477 (14)	0.0012 (11)	0.0201 (12)	−0.0042 (11)
C6	0.0393 (14)	0.0406 (13)	0.0474 (14)	0.0010 (11)	0.0131 (11)	−0.0014 (11)
C7	0.0432 (15)	0.0439 (15)	0.0511 (15)	0.0056 (12)	0.0177 (12)	0.0022 (12)
C8	0.0516 (17)	0.063 (2)	0.0511 (16)	0.0173 (15)	0.0190 (13)	0.0029 (14)
C9	0.072 (2)	0.095 (3)	0.067 (2)	0.016 (2)	0.0379 (19)	0.012 (2)
C10	0.098 (3)	0.133 (4)	0.068 (3)	0.027 (3)	0.045 (2)	0.019 (3)
C11	0.109 (4)	0.136 (5)	0.052 (2)	0.037 (3)	0.026 (2)	−0.002 (3)
C12	0.103 (4)	0.128 (5)	0.064 (2)	−0.003 (3)	0.013 (2)	−0.020 (3)
C13	0.078 (3)	0.092 (3)	0.062 (2)	−0.005 (2)	0.0208 (18)	−0.013 (2)
C14	0.0614 (19)	0.0409 (15)	0.0547 (16)	−0.0089 (13)	0.0229 (14)	−0.0029 (13)
C15	0.102 (3)	0.081 (3)	0.063 (2)	−0.010 (2)	0.034 (2)	0.0181 (19)
C16	0.0645 (19)	0.0386 (14)	0.0508 (14)	−0.0064 (14)	0.0210 (13)	−0.0058 (13)
C17	0.0482 (16)	0.0351 (14)	0.0570 (15)	−0.0053 (12)	0.0111 (12)	−0.0040 (12)
C18	0.0426 (15)	0.0369 (14)	0.0555 (15)	−0.0021 (11)	0.0091 (12)	0.0034 (12)
C19	0.0403 (14)	0.0319 (13)	0.0514 (14)	−0.0021 (10)	0.0129 (11)	0.0032 (11)
C20	0.0572 (17)	0.0313 (13)	0.0553 (15)	0.0038 (11)	0.0230 (13)	0.0046 (12)
C21	0.0399 (15)	0.0432 (15)	0.0593 (16)	−0.0034 (11)	0.0190 (12)	−0.0026 (12)
C22	0.0450 (16)	0.0408 (14)	0.0536 (15)	−0.0002 (11)	0.0163 (12)	0.0041 (12)
C23	0.0570 (17)	0.0487 (16)	0.0520 (15)	−0.0010 (14)	0.0200 (13)	0.0000 (13)
C24	0.067 (2)	0.0546 (18)	0.0584 (18)	−0.0084 (16)	0.0151 (15)	−0.0011 (15)
C25	0.100 (3)	0.066 (2)	0.066 (2)	−0.016 (2)	0.010 (2)	−0.0085 (19)
C26	0.127 (4)	0.076 (3)	0.060 (2)	−0.004 (3)	0.024 (2)	−0.017 (2)
C27	0.116 (4)	0.138 (5)	0.094 (3)	0.002 (4)	0.055 (3)	−0.042 (4)
C28	0.074 (3)	0.131 (4)	0.090 (3)	−0.013 (3)	0.040 (2)	−0.042 (3)
C29	0.0532 (17)	0.0525 (18)	0.0544 (16)	0.0037 (14)	0.0146 (13)	0.0132 (14)
C30	0.111 (4)	0.089 (3)	0.151 (5)	0.014 (3)	0.062 (4)	0.072 (4)

### Geometric parameters (Å, º)

**Table:**

Cl1—C1	1.791 (3)	C8—C13	1.374 (6)
Cl2—C16	1.782 (3)	C8—C9	1.386 (5)
O1—C2	1.439 (3)	C9—C10	1.378 (7)
O1—N1	1.442 (3)	C9—H9	0.9300
O2—C14	1.231 (4)	C10—C11	1.347 (9)
O3—C17	1.439 (4)	C10—H10	0.9300
O3—N6	1.445 (4)	C11—C12	1.352 (8)
O4—C29	1.226 (4)	C11—H11	0.9300
N1—C3	1.476 (4)	C12—C13	1.393 (6)
N1—H1N	0.85 (4)	C12—H12	0.9300
N2—C6	1.338 (4)	C13—H13	0.9300
N2—N3	1.340 (4)	C15—H15A	0.9600
N2—C5	1.471 (3)	C15—H15B	0.9600
N3—N4	1.307 (4)	C15—H15C	0.9600
N4—C7	1.359 (4)	C16—C17	1.523 (4)
N5—C14	1.316 (5)	C16—H16A	0.9700
N5—C15	1.461 (5)	C16—H16B	0.9700
N5—H5N	0.91 (4)	C17—C19	1.542 (4)
N6—C18	1.452 (4)	C17—H17	0.9800
N6—H6N	0.93 (5)	C18—C29	1.525 (4)
N7—C21	1.343 (4)	C18—C19	1.546 (4)
N7—N8	1.346 (3)	C18—H18	0.9800
N7—C20	1.459 (4)	C19—C20	1.528 (4)
N8—N9	1.305 (4)	C19—H19	0.9800
N9—C22	1.362 (4)	C20—H20A	0.9700
N10—C29	1.324 (5)	C20—H20B	0.9700
N10—C30	1.451 (5)	C21—C22	1.368 (4)
N10—H10N	0.78 (4)	C21—H21	0.9300
C1—C2	1.495 (4)	C22—C23	1.468 (4)
C1—H1A	0.9700	C23—C24	1.377 (5)
C1—H1B	0.9700	C23—C28	1.380 (5)
C2—C4	1.544 (4)	C24—C25	1.391 (5)
C2—H2	0.9800	C24—H24	0.9300
C3—C14	1.528 (4)	C25—C26	1.346 (7)
C3—C4	1.534 (4)	C25—H25	0.9300
C3—H3	0.9800	C26—C27	1.348 (8)
C4—C5	1.525 (4)	C26—H26	0.9300
C4—H4	0.9800	C27—C28	1.393 (7)
C5—H5A	0.9700	C27—H27	0.9300
C5—H5B	0.9700	C28—H28	0.9300
C6—C7	1.372 (4)	C30—H30A	0.9600
C6—H6	0.9300	C30—H30B	0.9600
C7—C8	1.471 (4)	C30—H30C	0.9600
			
C2—O1—N1	105.2 (2)	C8—C13—C12	119.8 (5)
C17—O3—N6	106.7 (2)	C8—C13—H13	120.1
O1—N1—C3	102.7 (2)	C12—C13—H13	120.1
O1—N1—H1N	98 (3)	O2—C14—N5	123.5 (3)
C3—N1—H1N	104 (3)	O2—C14—C3	121.1 (3)
C6—N2—N3	110.8 (2)	N5—C14—C3	115.4 (3)
C6—N2—C5	130.2 (2)	N5—C15—H15A	109.5
N3—N2—C5	119.0 (2)	N5—C15—H15B	109.5
N4—N3—N2	107.5 (2)	H15A—C15—H15B	109.5
N3—N4—C7	108.8 (2)	N5—C15—H15C	109.5
C14—N5—C15	122.3 (3)	H15A—C15—H15C	109.5
C14—N5—H5N	118 (3)	H15B—C15—H15C	109.5
C15—N5—H5N	119 (3)	C17—C16—Cl2	112.6 (2)
O3—N6—C18	104.3 (2)	C17—C16—H16A	109.1
O3—N6—H6N	107 (2)	Cl2—C16—H16A	109.1
C18—N6—H6N	109 (3)	C17—C16—H16B	109.1
C21—N7—N8	110.6 (2)	Cl2—C16—H16B	109.1
C21—N7—C20	128.3 (2)	H16A—C16—H16B	107.8
N8—N7—C20	121.1 (2)	O3—C17—C16	111.4 (2)
N9—N8—N7	107.0 (2)	O3—C17—C19	104.5 (2)
N8—N9—C22	109.7 (2)	C16—C17—C19	113.7 (2)
C29—N10—C30	123.5 (4)	O3—C17—H17	109.0
C29—N10—H10N	113 (3)	C16—C17—H17	109.0
C30—N10—H10N	123 (3)	C19—C17—H17	109.0
C2—C1—Cl1	111.4 (2)	N6—C18—C29	112.0 (3)
C2—C1—H1A	109.3	N6—C18—C19	107.3 (2)
Cl1—C1—H1A	109.3	C29—C18—C19	110.6 (2)
C2—C1—H1B	109.3	N6—C18—H18	109.0
Cl1—C1—H1B	109.3	C29—C18—H18	109.0
H1A—C1—H1B	108.0	C19—C18—H18	109.0
O1—C2—C1	108.1 (3)	C20—C19—C17	114.4 (2)
O1—C2—C4	105.6 (2)	C20—C19—C18	114.1 (2)
C1—C2—C4	116.0 (2)	C17—C19—C18	102.3 (2)
O1—C2—H2	109.0	C20—C19—H19	108.6
C1—C2—H2	109.0	C17—C19—H19	108.6
C4—C2—H2	109.0	C18—C19—H19	108.6
N1—C3—C14	107.5 (2)	N7—C20—C19	111.9 (2)
N1—C3—C4	106.6 (2)	N7—C20—H20A	109.2
C14—C3—C4	114.8 (2)	C19—C20—H20A	109.2
N1—C3—H3	109.3	N7—C20—H20B	109.2
C14—C3—H3	109.3	C19—C20—H20B	109.2
C4—C3—H3	109.3	H20A—C20—H20B	107.9
C5—C4—C3	113.1 (2)	N7—C21—C22	105.4 (3)
C5—C4—C2	115.5 (2)	N7—C21—H21	127.3
C3—C4—C2	101.7 (2)	C22—C21—H21	127.3
C5—C4—H4	108.7	N9—C22—C21	107.3 (3)
C3—C4—H4	108.7	N9—C22—C23	122.4 (3)
C2—C4—H4	108.7	C21—C22—C23	130.1 (3)
N2—C5—C4	109.9 (2)	C24—C23—C28	117.8 (3)
N2—C5—H5A	109.7	C24—C23—C22	121.5 (3)
C4—C5—H5A	109.7	C28—C23—C22	120.7 (3)
N2—C5—H5B	109.7	C23—C24—C25	120.2 (4)
C4—C5—H5B	109.7	C23—C24—H24	119.9
H5A—C5—H5B	108.2	C25—C24—H24	119.9
N2—C6—C7	104.9 (2)	C26—C25—C24	121.4 (4)
N2—C6—H6	127.5	C26—C25—H25	119.3
C7—C6—H6	127.5	C24—C25—H25	119.3
N4—C7—C6	107.9 (3)	C25—C26—C27	119.2 (4)
N4—C7—C8	122.1 (3)	C25—C26—H26	120.4
C6—C7—C8	130.0 (3)	C27—C26—H26	120.4
C13—C8—C9	118.0 (4)	C26—C27—C28	121.0 (5)
C13—C8—C7	121.2 (3)	C26—C27—H27	119.5
C9—C8—C7	120.8 (4)	C28—C27—H27	119.5
C10—C9—C8	120.6 (5)	C23—C28—C27	120.4 (5)
C10—C9—H9	119.7	C23—C28—H28	119.8
C8—C9—H9	119.7	C27—C28—H28	119.8
C11—C10—C9	121.1 (5)	O4—C29—N10	122.9 (3)
C11—C10—H10	119.5	O4—C29—C18	120.7 (3)
C9—C10—H10	119.5	N10—C29—C18	116.4 (3)
C10—C11—C12	119.2 (4)	N10—C30—H30A	109.5
C10—C11—H11	120.4	N10—C30—H30B	109.5
C12—C11—H11	120.4	H30A—C30—H30B	109.5
C11—C12—C13	121.4 (5)	N10—C30—H30C	109.5
C11—C12—H12	119.3	H30A—C30—H30C	109.5
C13—C12—H12	119.3	H30B—C30—H30C	109.5
			
C2—O1—N1—C3	−43.5 (3)	N1—C3—C14—O2	−94.4 (4)
C6—N2—N3—N4	−1.5 (4)	C4—C3—C14—O2	24.0 (4)
C5—N2—N3—N4	178.8 (3)	N1—C3—C14—N5	83.4 (3)
N2—N3—N4—C7	1.1 (4)	C4—C3—C14—N5	−158.2 (3)
C17—O3—N6—C18	39.2 (3)	N6—O3—C17—C16	86.8 (3)
C21—N7—N8—N9	0.0 (3)	N6—O3—C17—C19	−36.4 (3)
C20—N7—N8—N9	−179.4 (2)	Cl2—C16—C17—O3	57.2 (3)
N7—N8—N9—C22	0.3 (3)	Cl2—C16—C17—C19	175.0 (2)
N1—O1—C2—C1	161.7 (2)	O3—N6—C18—C29	96.0 (3)
N1—O1—C2—C4	36.9 (3)	O3—N6—C18—C19	−25.6 (3)
Cl1—C1—C2—O1	67.3 (3)	O3—C17—C19—C20	143.0 (2)
Cl1—C1—C2—C4	−174.4 (2)	C16—C17—C19—C20	21.3 (4)
O1—N1—C3—C14	156.5 (2)	O3—C17—C19—C18	19.1 (3)
O1—N1—C3—C4	32.9 (3)	C16—C17—C19—C18	−102.6 (3)
N1—C3—C4—C5	−135.3 (2)	N6—C18—C19—C20	−120.2 (3)
C14—C3—C4—C5	105.8 (3)	C29—C18—C19—C20	117.4 (3)
N1—C3—C4—C2	−10.8 (3)	N6—C18—C19—C17	4.0 (3)
C14—C3—C4—C2	−129.7 (2)	C29—C18—C19—C17	−118.5 (3)
O1—C2—C4—C5	107.6 (3)	C21—N7—C20—C19	116.5 (3)
C1—C2—C4—C5	−12.1 (4)	N8—N7—C20—C19	−64.3 (4)
O1—C2—C4—C3	−15.3 (3)	C17—C19—C20—N7	−176.9 (2)
C1—C2—C4—C3	−135.0 (3)	C18—C19—C20—N7	−59.5 (3)
C6—N2—C5—C4	111.7 (3)	N8—N7—C21—C22	−0.3 (3)
N3—N2—C5—C4	−68.7 (3)	C20—N7—C21—C22	179.0 (3)
C3—C4—C5—N2	−73.6 (3)	N8—N9—C22—C21	−0.5 (4)
C2—C4—C5—N2	169.7 (2)	N8—N9—C22—C23	175.9 (3)
N3—N2—C6—C7	1.2 (3)	N7—C21—C22—N9	0.5 (3)
C5—N2—C6—C7	−179.2 (3)	N7—C21—C22—C23	−175.6 (3)
N3—N4—C7—C6	−0.4 (4)	N9—C22—C23—C24	175.4 (3)
N3—N4—C7—C8	179.8 (3)	C21—C22—C23—C24	−9.0 (5)
N2—C6—C7—N4	−0.5 (3)	N9—C22—C23—C28	−7.6 (5)
N2—C6—C7—C8	179.3 (3)	C21—C22—C23—C28	168.0 (4)
N4—C7—C8—C13	−148.7 (4)	C28—C23—C24—C25	−2.0 (6)
C6—C7—C8—C13	31.5 (5)	C22—C23—C24—C25	175.1 (3)
N4—C7—C8—C9	33.0 (5)	C23—C24—C25—C26	1.6 (6)
C6—C7—C8—C9	−146.8 (4)	C24—C25—C26—C27	−1.2 (8)
C13—C8—C9—C10	−0.5 (6)	C25—C26—C27—C28	1.3 (10)
C7—C8—C9—C10	177.9 (4)	C24—C23—C28—C27	2.1 (8)
C8—C9—C10—C11	−0.3 (8)	C22—C23—C28—C27	−175.0 (5)
C9—C10—C11—C12	0.7 (8)	C26—C27—C28—C23	−1.8 (10)
C10—C11—C12—C13	−0.4 (9)	C30—N10—C29—O4	−1.5 (7)
C9—C8—C13—C12	0.8 (6)	C30—N10—C29—C18	177.9 (5)
C7—C8—C13—C12	−177.5 (4)	N6—C18—C29—O4	−172.9 (3)
C11—C12—C13—C8	−0.4 (8)	C19—C18—C29—O4	−53.2 (4)
C15—N5—C14—O2	3.2 (6)	N6—C18—C29—N10	7.6 (4)
C15—N5—C14—C3	−174.6 (3)	C19—C18—C29—N10	127.3 (3)

### Hydrogen-bond geometry (Å, º)

Cg2 is the centroid of the triazole ring N2–N4/C6/C7 in molecule *A*.

**Table:**

*D*—H···*A*	*D*—H	H···*A*	*D*···*A*	*D*—H···*A*
N10—H10*N*···N6	0.78 (4)	2.21 (4)	2.668 (4)	118 (4)
N5—H5*N*···O4	0.91 (4)	2.02 (4)	2.925 (4)	173 (4)
C6—H6···N9	0.93	2.37	3.275 (4)	164
C1—H1*B*···O2^i^	0.97	2.36	3.208 (4)	146
C5—H5*B*···O2^i^	0.97	2.47	3.432 (4)	171
C16—H16*A*···N3^ii^	0.97	2.37	3.335 (4)	176
C2—H2···*Cg*2^iii^	0.95	2.90	3.806 (3)	154

Symmetry codes: (i) −*x*, *y*+1/2, −*z*+1; (ii) *x*+1, *y*, *z*; (iii) −*x*, *y*−1/2, −*z*+1.
